# Dilaton-induced open quantum dynamics

**DOI:** 10.1140/epjc/s10052-023-11939-4

**Published:** 2023-08-31

**Authors:** Christian Käding, Mario Pitschmann, Caroline Voith

**Affiliations:** Technische Universität Wien, Atominstitut, Stadionallee 2, 1020 Vienna, Austria

## Abstract

In modern cosmology, scalar fields with screening mechanisms are often used as explanations for phenomena like dark energy or dark matter. Amongst a zoo of models, the environment dependent dilaton, screened by the Polyakov–Damour mechanism, is one of the least constrained ones. Using recently developed path integral tools for directly computing reduced density matrices, we study the open quantum dynamics of a probe, modelled by another real scalar field, induced by interactions with an environment comprising fluctuations of a dilaton. As the leading effect, we extract a correction to the probe’s unitary evolution, which can be observed as a frequency shift. Assuming the scalar probe to roughly approximate a cold atom in matter wave interferometry, we show that comparing the predicted frequency shifts in two experimentally distinct setups has the potential to exclude large parts of the dilaton parameter space.

## Introduction

In modern cosmology, scalar-tensor theories of gravity [1], in which scalar fields are coupled to the gravitational metric tensor, are often proposed as potential solutions for the problems of dark matter and dark energy, see Refs.  [2, 3] for an overview. In many cases, such scalar-tensor theories lead to a universal coupling of the scalar to the trace $T^\mu _{\,\,\,\mu }$ of the matter energy-momentum tensor. Consequently, these theories predict the existence of gravity-like fifth forces. However, to date, we did not observe any additional fundamental forces beyond the four known ones, which is reflected in strong Solar System constraints [4–6].

A phenomenologically interesting way of explaining the apparent absence of fifth forces in our Solar System is via a so-called screening mechanism. Such mechanisms can arise in non-linear scalar field theories, and induce a screening of the scalar’s fifth force, i.e.  render it to be very weak, in environments with large values of $T^\mu _{\,\,\,\mu }$, for example, for high densities $\rho ^{\text {ext}} = -T^\mu _{\,\,\,\mu }$ in dust-dominated regions like our Solar System. By now, there are several scalar models which exhibit a screening mechanism [7, 8]. Amongst the most prominent so-called screened scalar fields are: the chameleon [9, 10]; the symmetron [11–18], whose fifth force has recently been studied as an alternative to particle dark matter [19–22]; the environment dependent dilaton [13, 23–28]; and the galileon [29–31]. Many of these models have already been or are proposed to be tested in a plethora of experiments, see, for example, Refs.  [7, 32–48]. In addition, in recent years, research programs were initiated to study screened scalars as quantum fields [49–52], and it was even suggested to investigate screened scalar-tensor theories in analogue gravity simulations [53].

Reference  [50] presented the idea for using open quantum dynamical effects [54] like frequency shifts or decoherence [55], which might be induced by interactions with an environment comprising light scalar fluctuations, for further constraining chameleon screened scalar fields in atom interferometry experiments. Besides presenting phenomenological research, Ref.  [50] also introduced a novel and powerful theoretical tool, namely a practicable and first principle-based method for deriving quantum master equations for reduced density matrices in scalar quantum field theory. This method relies on the Schwinger–Keldysh formalism [56, 57] and the Feynman–Vernon influence functional [58]. Based on the work in Ref.  [50], Ref.  [59] developed a way of circumventing master equations by directly computing reduced density matrix elements with influence functionals. In addition, Ref.  [60] derived a similar technique for computing total density matrices in interacting quantum field theories.

In this article, we look at the environment dependent dilaton screened scalar field as a yet barely constrained model. Following the approach made in Ref.  [50], we study the open quantum dynamics of a probe real scalar field $\phi$ when interacting with an environment comprising fluctuations $\chi$ of a dilaton field *X*. More precisely, we use the scalar $\phi$ as a proxy for an atom in an atom interferometry experiment (or any other object in a matter interferometer, e.g., neutrons) and, applying the formalism from Ref.  [59], check how its reduced density matrix evolves from an initial time 0 to a final time *t* due to the hypothetical interaction with a dilaton. We then read off the most dominant open quantum dynamical effect induced in the probe system $\phi$ in order to get an estimate of the dilaton parameter space that could be constrained experimentally this way.

Certainly, using a scalar field $\phi$ as a proxy for a more complex object like an atom has its shortcomings. However, since the dilaton only couples to the trace of the atom’s energy-momentum tensor, a scalar field is a rough but, in this case, suitable first approximation. In order to improve this approximation, we consider the probe system being restricted to a single-particle subspace and ignore all $\phi$-loop diagrams, which naturally appear in a scalar quantum field theory, but are unrealistic for a complex and stable object like a cold atom.

Providing a glance at the full potential that open quantum dynamical effects can have for constraining physics beyond the standard models of particles and cosmology, this article presents a first application of Ref.  [59], and motivates future studies with more realistic and sophisticated probe models.

The article is structured as follows: at first, we review the environment dependent dilaton model in Sect.  2. Subsequently, we take the required formula from Ref.  [59], discuss and evaluate it in Sect.  3, and then use the results for estimating constraints for the dilaton parameter space in Sect.  4. Finally, in Sect.  5, we draw our conclusions.

## Environment dependent dilatons

Dilatons originate from string theory [13, 24, 25], but have found applications in cosmology [23, 27] and were shown, similar to symmetrons, to be subject to the Polyakov–Damour mechanism [13], which coins them to be environment dependent [26] and therefore a screened scalar field model.

The environment dependent dilaton has an effective potential [3]

1 $$\begin{aligned} V_{\text {eff}}(\varphi ;\rho ^{\text {ext}})= & {} \bar{V_0} \, e^{-\varphi /M_{Pl}} + \frac{(\varphi -\varphi _*)^2}{2 \mathcal {M}^2}\rho ^{\text {ext}}, \end{aligned}$$

where $\bar{V_0}$ is a constant potential with a decreasing exponential function $\exp (-\varphi /M_{Pl})$ as required by the strong coupling limit of string theory. Here, $M_{Pl}$ denotes the Planck mass, $\mathcal {M}$ is a coupling constant with dimension of a mass, for which we require $\varphi \ll \mathcal {M}$, and $\varphi _*$ is a constant field value around which the coupling of the dilaton to matter becomes feeble or even vanishes entirely for $\varphi =\varphi _*$. This decoupling is the essence of the Polyakov–Damour mechanism and leads to a suppression of the dilaton fifth force.

The fact that a larger value of $\rho ^{\text {ext}}$ actually corresponds to a weaker coupling to matter can more easily be seen when substituting $\lambda X:= \varphi -\varphi _*$ into Eq.  (1). Introducing

2 $$\begin{aligned} V(X) :=\, V_0 \,e^{-\lambda X/M_{Pl}}:= & {} \bar{V_0} \, e^{-(\lambda X+\varphi _*)/M_{Pl}} \end{aligned}$$

with $\lambda$ being a dimensionless coupling constant, we find [28]

3 $$\begin{aligned} V_{\text {eff}}(X;\rho ^{\text {ext}})= & {} V_0 \,e^{-\lambda X/M_{Pl}} + \frac{A_2 \rho ^{\text {ext}}}{2M_{Pl}^2} X^2 , \end{aligned}$$

where a dimensionless constant $A_2:= \lambda ^2 M_{Pl}^2/\mathcal {M}^2$ was introduced that has to fulfill $A_2 \gg 1$ in order to circumvent existing Solar System-based constraints on scalar fifth forces [16]. From Eq.  (3) we find the dilaton vacuum expectation value (vev) as [28]

4 $$\begin{aligned} \langle X \rangle= & {} \frac{M_{Pl}}{\lambda } W\left( \frac{\lambda ^2 V_0}{A_2 \rho ^{\text {ext}}} \right) \end{aligned}$$

with the Lambert *W*-function

5 $$\begin{aligned} W(x)= & {} \sum _{n=1}^{\infty } \frac{(-n)^{n-1}}{n!} x^n. \end{aligned}$$

Since $X = \langle X \rangle + \chi$, where $\chi$ is a small perturbation, Eq.  (4) lets us conclude that *X* decreases when $\rho ^{\text {ext}}$ increases. Consequently, the leading $\langle X \rangle ^2$-term in Eq.  (3) becomes smaller for larger values of $\rho ^{\text {ext}}$, which indicates a decoupling between dilaton and matter, i.e.  a screening of the fifth force.

As a scalar-tensor theory, the dilaton is coupled to the gravitational metric tensor via $\tilde{g}_{\mu \nu } = A^2(X) g_{\mu \nu },$ where $\tilde{g}$ and *g* denote the metric in the Jordan and Einstein frame [1], respectively, and the conformal factor is given by

6 $$\begin{aligned} A(X)= & {} 1 + \frac{A_2}{2M_{Pl}^2} X^2. \end{aligned}$$

The total Einstein frame action describing gravity, the scalar *X* and matter is given by [9, 10]

7 $$\begin{aligned} S= & {} \int d^4x \sqrt{-g} \left[ \frac{1}{2} M_{Pl}^2 R - \frac{1}{2} g^{\mu \nu } \partial _{\mu }X\partial _{\nu }X - V(X) \right] \nonumber \\{} & {} +\int d^4x \sqrt{-g} A^4(X) \tilde{\mathcal {L}}_M (\tilde{\phi }, A^2(X) g_{\mu \nu }) ,\,\,\,\,\,\, \end{aligned}$$

where the last term describes the dynamics of the Jordan frame matter field $\tilde{\phi }$, which in our case is a real scalar, and its interactions with the dilaton. Here, the potential *V*(*X*) is the self-interaction of *X* given by Eq.  (2), and for the Jordan frame matter Lagrangian we use

8 $$\begin{aligned} \tilde{\mathcal {L}}_M= & {} -\frac{1}{2} \tilde{g}^{\mu \nu } \partial _{\mu } \tilde{\phi } \partial _{\nu } \tilde{\phi } - \frac{1}{2} \tilde{M}^2 \tilde{\phi }^2. \end{aligned}$$

Since we will have to work with the dilaton perturbatively if we later want to discuss it in the formalism presented in Ref.  [59], we are required to assume $\lambda X/M_{Pl} \ll 1$, such that we can expand

9 $$\begin{aligned} V(X)\approx & {} V_0 \left[ 1 -\frac{\lambda }{M_{Pl}}X + \frac{1}{2} \frac{\lambda ^2 }{M_{Pl}^2}X^2 + \mathcal {O}\bigg ( \frac{\lambda ^3 }{M_{Pl}^3}X^3\bigg ) \right] .\nonumber \\ \end{aligned}$$

Following the general procedure outlined in Ref.  [50] for Eqs.  (6)–(9), while only keeping operators of dimension 4 or lower, we can derive Einstein frame free actions for the matter field $\phi$ and the dilaton fluctuation $\chi$ as well as an interaction action between both types of fields:

10 $$\begin{aligned} S_{\phi }[\phi ]:= & {} \int _x \left[ - \frac{1}{2} g^{\mu \nu } \partial _{\mu } \phi \partial _{\nu } \phi - \frac{1}{2} M^2 \phi ^2 \right] , \end{aligned}$$

11 $$\begin{aligned} S_{\chi }[\chi ]:= & {} \int _x \left[ - \frac{1}{2} g^{\mu \nu } \partial _{\mu } \chi \partial _{\nu } \chi - \frac{1}{2} m^2 \chi ^2 \right] , \end{aligned}$$

12 $$\begin{aligned} S_{\text {int}}[\phi , \chi ]:= & {} \int _{x \in \Omega _t} \left[ -\frac{1}{2} \alpha _1 M\chi \phi ^2 -\frac{1}{4}\alpha _2 \chi ^2 \phi ^2 \right] , \end{aligned}$$

where, using $g_{\mu \nu } \equiv \eta _{\mu \nu }$ from now on,

13 $$\begin{aligned} \int _x:= & {} \int d^4x , \end{aligned}$$

the masses are

14 $$\begin{aligned} M^2:= & {} \left( 1+\frac{A_2}{M_{Pl}^2} \langle X \rangle ^2 \right) \tilde{M}^2\, \nonumber \\ m^2\approx & {} \frac{1}{M_{Pl}^2} \left( V_0 \lambda ^2 + A_2 \rho ^{\text {ext}} \right) , \end{aligned}$$

the coupling constants take on the forms

15 $$\begin{aligned} \alpha _1:= & {} 2M \frac{A_2}{M_{Pl}^2} \langle X \rangle \left( 1-\frac{A_2}{M_{Pl}^2} \langle X \rangle ^2 \right) , \quad \alpha _2 :=\, \frac{M}{\langle X \rangle } \alpha _1 ,\nonumber \\ \end{aligned}$$

and we restrict integrations over spacetime coordinates to  $\Omega _t:= [0,t] \times \mathbb {R}$  since we are only interested in the finite interval between initial time 0 and final time *t*. Note that at the order considered in the expansion made in Eq.  (9) the dilaton does not self-interact and, therefore, we do not need to consider a self-interaction action for $\chi$.

## Open quantum dynamics

Most realistic quantum systems should be treated as open, i.e.  as interacting with one or several environments. Such interactions can induce open quantum dynamical effects like frequency (or phase) shifts or decoherence in a system. While the theory of open quantum systems naturally finds many applications in non-relativistic quantum physics, see e.g.  Refs. [61–67], it also receives increased attention in quantum field theory [50, 68–76], and adjacent areas like Early Universe cosmology [77–89], black holes [90–96], or heavy-ion physics [97–106].

Quantum systems, especially open ones, are often described by density operators $\hat{\rho }(t)$, which are advantageous over a wave function description since they can not only describe pure but also mixed states. This is necessary in order to fully capture phenomena like decoherence. In case of an open quantum system, we usually work with reduced density operators, which are obtained by tracing out the environmental degrees of freedom.

Projecting a (reduced) density operator into a basis, for example, a single-particle momentum basis as we will use in this article, gives elements of a density matrix:

16 $$\begin{aligned} \rho (\textbf{p};\textbf{p}';t)= & {} \langle \textbf{p}|\hat{\rho }(t) |\textbf{p}'\rangle . \end{aligned}$$

The time evolution of density matrices can be described by quantum master equations. However, such equations are often analytically intricate or even impossible to solve. In order to avoid such complications, using the technology presented in Ref.  [50], Ref.  [59] developed a formalism that enables us to directly compute reduced density matrices in terms of the Feynman–Vernon influence functional [58], which itself is based on the Schwinger–Keldysh formalism [56, 57].

In this article, we consider an open quantum system $\phi$, which is a proxy for an atom (or another, compared to the dilaton relatively heavy matter particle like a neutron) with zero temperature in order to justify a restriction to the single-particle subspace. As was pointed out in Refs.  [50, 59], in this case, contractions of the system field can only give rise to Feynman and Dyson propagators:1718where

19 $$\begin{aligned} \int _{k}:= & {} \int \frac{d^4 k}{(2\pi )^4} , \end{aligned}$$

and $+$ and − denote the positive and negative branches of the Schwinger–Keldysh closed time path, respectively.

The system is interacting with an environment comprised of dilaton fluctuations $\chi$. As was also done in Ref.  [50] for the chameleon, we assume the dilaton to have a, in general, non-zero temperature *T*, for example, due to thermalization with the walls of a vacuum chamber in an atom interferometry experiment. This means, open system and environment are out of thermal equilibrium in our discussion. Since environmental degrees of freedom can be contracted for any combination of $+$ and − [50, 59], the dilaton can give Feynman and Dyson, but also Wightman propagators:20212223where

24 $$\begin{aligned} f(k^0):= & {} \frac{1}{e^{\beta k^0}-1} \,=\,-[1+f(-k^0)] \end{aligned}$$

is the Bose-Einstein distribution function with $\beta = 1/T$ being the inverse temperature.

Following Ref.  [59], the reduced density matrix element for $\phi$ at time *t* in the single-particle momentum subspace, under the assumption that the system’s particle number does not change in the interval [0, *t*], can be obtained by evaluating

25 $$\begin{aligned}{} & {} \rho (\textbf{p};\textbf{p}';t) = \lim _{\begin{array}{c} x^{0(\prime )}\,\rightarrow \, t^{+}\\ y^{0(\prime )}\,\rightarrow \, 0^- \end{array}} \int _{\textbf{k}\textbf{k}'} \frac{\rho (\textbf{k};\textbf{k}';0) }{(2 E_{\textbf{k}}^\phi )(2 E_{\textbf{k}'}^\phi )} \nonumber \\{} & {} \quad \times \int _{\textbf{x}\textbf{x}'\textbf{y}\textbf{y}'} e^{-\textrm{i}(\textbf{p}\cdot \textbf{x}-\textbf{p}' \cdot \textbf{x}')+\textrm{i}(\textbf{k}\cdot \textbf{y}-\textbf{k}'\cdot \textbf{y}')}\nonumber \\{} & {} \quad \times \partial _{x^0,E^\phi _{\textbf{p}}} \partial _{x^{0\prime },E^\phi _{\textbf{p}'}}^*\partial _{y^0,E^\phi _{\textbf{k}}}^*\partial _{y^{0\prime },E^\phi _{\textbf{k}'}} \nonumber \\{} & {} \quad \times \int \mathcal {D}\phi ^{\pm } e^{\textrm{i}\left\{ S_{\phi }[\phi ^+]-S_{\phi }[\phi ^-]\right\} }\phi ^+_x\phi ^-_{x'}\nonumber \\{} & {} \quad \times \mathcal {F}[\phi ^\pm ;t]\phi ^{+}_y\phi ^{-}_{y'} , \end{aligned}$$

where the Feynman–Vernon influence functional

26 $$\begin{aligned} \mathcal {F}[\phi ^\pm ;t]= & {} \left\langle e^{\textrm{i}\big \{ S_\text {int}[\phi ^+,\chi ^+;t]- S_\text {int}[\phi ^-,\chi ^-;t] \big \}} \right\rangle _\chi \end{aligned}$$

is given in terms of the expectation value

27 $$\begin{aligned} \langle A[\chi ^{a}]\rangle _\chi:= & {} \int d\chi ^{\pm }_t d\chi ^{\pm }_0 \delta (\chi _t^+-\chi _t^-)\rho _\chi [\chi ^{\pm }_0;0]\nonumber \\{} & {} \times \int ^{\chi ^{\pm }_t}_{\chi ^{\pm }_0} \mathcal {D}\chi ^{\pm } A[\chi ^{a}]e^{\textrm{i}\left\{ S_{\chi }[\chi ^+]-S_{\chi }[\chi ^-]\right\} } \end{aligned}$$

with $\rho _\chi [\chi ^{\pm }_0;0]$ denoting the initial environmental density matrix under the assumption that system and environment were initially uncorrelated. The index of a field, e.g.  *t* on $\chi _t$, labels the time slice on which the field eigenstate was taken.

We expand the Feynman–Vernon influence functional up to second order in the coupling constants $\alpha _1$ and $\alpha _2$, and find

28 $$\begin{aligned} \mathcal {F}[\phi ^\pm ;t]= & {} 1 - \textrm{i} \frac{\alpha _2}{4} \sum _{a=\pm } a \int _x (\phi ^a_x)^2 \Delta ^\textrm{F}_{xx} \nonumber \\{} & {} - \frac{1}{2} \sum _{a,b=\pm } ab \int _{xy} \biggl [ \frac{\alpha _1^2 M^2}{4} (\phi ^a_x)^2 (\phi ^b_y)^2 \Delta ^{ab}_{xy} \nonumber \\{} & {} + \frac{\alpha _2^2}{16} (\phi ^a_x)^2 (\phi ^b_y)^2 \left( \Delta ^\textrm{F}_{xx} \Delta ^\textrm{F}_{yy} +2(\Delta ^{ab}_{xy})^2 \right) \bigg ]\nonumber \\{} & {} + \mathcal {O}\left( \alpha ^3_{1,2}\right) . \end{aligned}$$

Substituting Eq.  (28) into Eq.  (25), using Wick’s theorem [107], and dropping every term containing $\phi$-loops, we obtain

29 $$\begin{aligned}{} & {} \rho (\textbf{p}; \textbf{p}';t) = \lim _{\begin{array}{c} x^{0(\prime )}\rightarrow t^+\\ y^{0(\prime )} \rightarrow 0^- \end{array}} \int _{\textbf{k}\textbf{k}'} \frac{\rho (\textbf{k};\textbf{k}';0) }{(2 E_{\textbf{k}}^\phi )(2 E_{\textbf{k}'}^\phi )} \nonumber \\{} & {} \quad \times \int _{\textbf{x}\textbf{x}'\textbf{y}\textbf{y}'} e^{-\textrm{i} (\textbf{p}\cdot \textbf{x}-\textbf{p}'\cdot \textbf{x}')+\textrm{i} (\textbf{k}\cdot \textbf{y}-\textbf{k}'\cdot \textbf{y}')}\nonumber \\{} & {} \quad \partial _{x^0,E_\textbf{p}^\phi } \partial ^*_{x^{0\prime },E_{\textbf{p}'}^\phi } \partial ^*_{y^0,E_\textbf{k}^\phi } \partial _{y^{0\prime },E_{\textbf{k}'}^\phi } \nonumber \\{} & {} \quad \times \Bigg \{ D^\textrm{F}_{xy}D^\textrm{D}_{x'y'} \nonumber \\{} & {} \quad - \textrm{i} \frac{\alpha _2}{2} \int _z \left[ D^\textrm{F}_{xz}D^\textrm{F}_{zy}D^\textrm{D}_{x'y'} - (x,y \longleftrightarrow x',y')^*\right] \Delta ^\textrm{F}_{zz} \nonumber \\{} & {} \quad - \frac{\alpha _1^2 M^2}{8} \int _{zz'} \bigg [ \Big ( D^\textrm{D}_{x'y'}\big (8 D^\textrm{F}_{xz} D^\textrm{F}_{zz'} D^\textrm{F}_{z'y} + 2 D^\textrm{F}_{xy} D^\textrm{F}_{zz'} D^\textrm{F}_{zz'} \big ) \Delta ^\textrm{F}_{zz'} \nonumber \\{} & {} \quad +(x,y \longleftrightarrow x',y')^*\Big ) \nonumber \\{} & {} \quad - \big (8 D^\textrm{F}_{xz} D^\textrm{F}_{zy} D^\textrm{D}_{x'z'} D^\textrm{D}_{z'y'} + 4 D^\textrm{F}_{xy} D^\textrm{F}_{zz} D^\textrm{D}_{x'z'} D^\textrm{D}_{z'y'} \big ) \Delta ^{+-}_{zz'} \bigg ] \nonumber \\{} & {} \quad - \frac{\alpha _2^2}{32} \int _{zz'} \bigg [ \Big ( D^\textrm{D}_{x'y'}\big (8 D^\textrm{F}_{xz} D^\textrm{F}_{zz'} D^\textrm{F}_{z'y} + 2 D^\textrm{F}_{xy} D^\textrm{F}_{zz'} D^\textrm{F}_{zz'} \big ) \nonumber \\{} & {} \quad \big (\Delta ^\textrm{F}_{zz}\Delta ^\textrm{F}_{z'z'}+ 2(\Delta ^\textrm{F}_{zz'})^2 \big ) +(x,y \longleftrightarrow x',y')^*\Big ) \nonumber \\{} & {} \quad - \big (8 D^\textrm{F}_{xz} D^\textrm{F}_{zy} D^\textrm{D}_{x'z'} D^\textrm{D}_{z'y'} \nonumber \\{} & {} \quad + 4 D^\textrm{F}_{xy} D^\textrm{F}_{zz} D^\textrm{D}_{x'z'} D^\textrm{D}_{z'y'} \big ) \big (\Delta ^\textrm{F}_{zz}\Delta ^\textrm{F}_{z'z'} + 2(\Delta ^{+-}_{zz'})^2 \big ) \bigg ] \Bigg \} . \end{aligned}$$

**Fig. 1:**
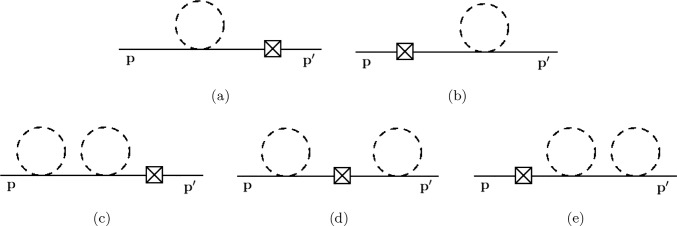
Diagrammatic representation of of the last two terms in the square brackets in Eq.  (30); solid/dashed lines represent $\phi$-/$\chi$-propagators. Crossed boxes depict insertions of the initial density matrix

Next, we evaluate the integrals in Eq.  (29). The full result of this computation can be found in Appendix A. However, for the present discussion we focus only on the following terms:

30 $$\begin{aligned}{} & {} \rho (\textbf{p}; \textbf{p}';t) = \rho (\textbf{p}; \mathbf {p'};0) e^{-\textrm{i}(E_{\textbf{p}}^{\phi } - E_{\mathbf {p'}}^{\phi }) t} \nonumber \\{} & {} \quad \times \left[ 1 - \frac{\textrm{i} \alpha _2}{4} \left( \frac{1}{E_{\textbf{p}}^{\phi }} - \frac{1}{E_{\mathbf {p'}}^{\phi }} \right) t \Delta ^\textrm{F}_{zz}\right. \nonumber \\{} & {} \left. \quad - \frac{\alpha _2^2}{32} \left( \frac{1}{E_{\textbf{p}}^{\phi }} - \frac{1}{E_{\mathbf {p'}}^{\phi }} \right) ^2t^2 \Delta ^\textrm{F}_{zz} \Delta ^\textrm{F}_{z'z'} \right] + \mathcal {O}\left( \alpha ^2_{1,2}\right) . \end{aligned}$$

A diagrammatic depiction of the last two terms in the square brackets can be found in Fig.  1.

Following Ref.  [50], we deal with the tadpoles in Eq.  (30) by adding a counter term

31 $$\begin{aligned} \delta S_{\text {int}}[\phi , \chi ]:= & {} \ \frac{\alpha _2}{4} \int _{x} \Delta ^{F(T=0)}_{xx} \phi ^2 \end{aligned}$$

to the interaction action in Eq.  (12), where $(T=0)$ indicates the temperature-independent part of the $\chi$-propagator. The thermal part

32 $$\begin{aligned} \Delta ^{\textrm{F}(T\ne 0)}_{xx}= & {} \int _k 2\pi f(|k^0|) \delta (k^2+m^2) \nonumber \\= & {} \frac{T^2}{2\pi ^2} \int _{m/T}^\infty d\xi \frac{\sqrt{\xi ^2-(\frac{m}{T})^2}}{e^\xi -1} \end{aligned}$$

is by default finite in the ultraviolet. Using this, and identifying the terms in the square brackets of Eq.  (30) as the expansion of an exponential function, we find

33 $$\begin{aligned}{} & {} \rho (\textbf{p}; \textbf{p}';t) = \rho (\textbf{p}; \mathbf {p'};0) \exp \nonumber \\{} & {} \quad \times \left\{ -\textrm{i}\Bigg [E_{\textbf{p}}^{\phi } - E_{\mathbf {p'}}^{\phi } + \frac{\alpha _2}{4} \left( \frac{1}{E_{\textbf{p}}^{\phi }} - \frac{1}{E_{\mathbf {p'}}^{\phi }} \right) \Delta ^{\textrm{F}(T\ne 0)}_{zz}\Bigg ] t\right\} \nonumber \\{} & {} \quad + \mathcal {O}\left( \alpha ^2_{1,2}\right) . \end{aligned}$$

Now we recall that we performed all computations with the rescaled mass *M* as given in Eq.  (14). However, cp.  with Ref.  [50], experiments are actually sensitive to the absolute mass $\tilde{M}$. Rewriting Eq.  (33), we obtain

34 $$\begin{aligned} \rho (\textbf{p}; \textbf{p}';t)= & {} \rho (\textbf{p}; \mathbf {p'};0) \exp \left\{ -\textrm{i}\left[ \tilde{E}_\textbf{p}^\phi - \tilde{E}_{\textbf{p}'}^\phi \right. \right. \nonumber \\{} & {} \left. \left. + \tilde{M}^2 \frac{A_2}{2M_{Pl}^2} \left( \langle X\rangle ^2 + \Delta ^{\textrm{F}(T\ne 0)}_{zz} \right) \right. \right. \nonumber \\{} & {} \times \left. \left. \left( \frac{1}{\tilde{E}_\textbf{p}^\phi } - \frac{1}{\tilde{E}_{\textbf{p}'}^\phi } \right) \right] t\right\} + \mathcal {O}\left( \alpha ^2_{1,2}\right) , \end{aligned}$$

where $\tilde{E}_\textbf{p}^\phi =\sqrt{\textbf{p}^2+\tilde{M}^2}$, we replaced $\alpha _2$ by Eq.  (15), and neglected the term $\sim \mathcal {O}(A_2^2\tilde{M}^2 \langle X \rangle ^2/M_{Pl}^4)$. What we found in Eq.  (34) is a first order correction to the unitary evolution term, i.e.  a phase shift $\Delta u \cdot t$ with a frequency shift $\Delta u$. Other effects like de-/recoherence and momentum diffusion first appear at second order, see Appendix 1, and are therefore expected to be subdominant.

**Fig. 2:**
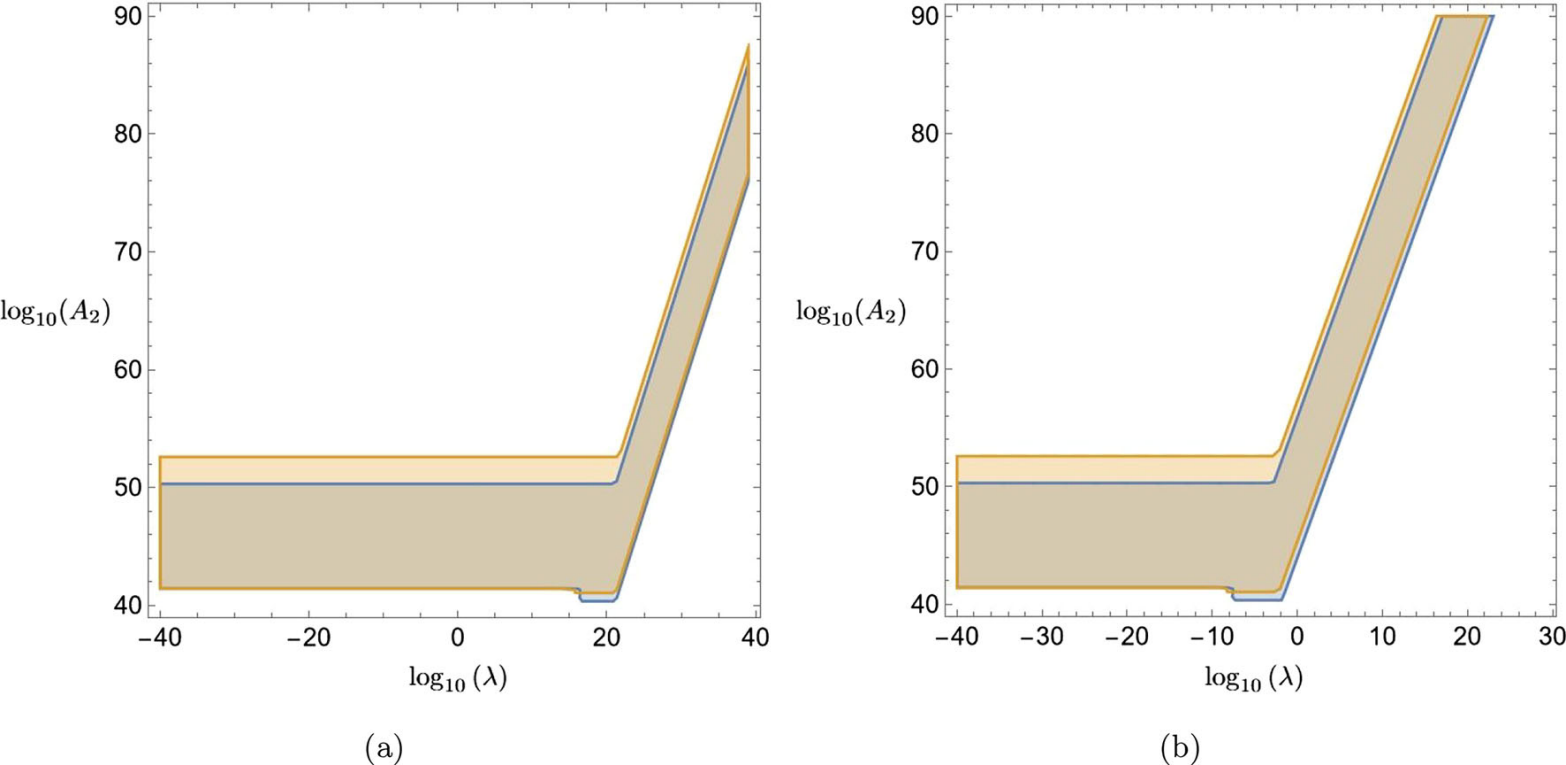
Exclusion plots for the environment dependent dilaton $(\lambda ,A_2)$-parameter space resulting from differences in frequency shifts $u(P_1,T_2;P_1,T_1)$ (blue) and $u(P_1,T_4;P_1,T_1)$ (orange); **a** $V_0 = 1\,\text {eV}^4$, **b** $V_0 = 1\,(\text {MeV})^4$

## Predicted constraints

Atom interferometry experiments have successfully been used to constrain a variety of screened scalar field models [35, 36, 38, 39, 41, 42, 44]. As was also done in Ref.  [50], we now estimate whether the frequency shift found in Eq.  (34) could potentially be observed in an atom interferometer. Since such experiments use low-energy atoms, we make a non-relativistic approximation $\tilde{M}^2 \gg \textbf{p}^2$, such that

35 $$\begin{aligned} \frac{1}{\tilde{E}_\textbf{p}^\phi }\approx & {} \frac{1}{\tilde{M}} \left( 1- \frac{\textbf{p}^2}{2\tilde{M}^2}\right) ,\quad \frac{1}{\tilde{E}_\textbf{p}^\phi } - \frac{1}{\tilde{E}_{\textbf{p}'}^\phi } \,\approx \,\frac{v^2}{2\tilde{M}} \end{aligned}$$

with $v:= \frac{||\textbf{p}|-|\textbf{p}'||}{\tilde{M}}$ being the speed difference between the two atomic states. Substituting this into Eq.  (34), gives for the frequency shift:

36 $$\begin{aligned} \Delta u:= & {} \tilde{M} \frac{A_2}{4M_{Pl}^2} \left[ \langle X\rangle ^2 + \Delta ^{\textrm{F}(T\ne 0)}_{zz} \right] v^2 . \end{aligned}$$

For the estimation we choose commonly used values for the parameters of the experiment: a vacuum chamber with radius $L = 10$ cm [35, 38], and a Rubidium-87 atom with a mass of $\tilde{M}=87\, m_u$ [44], where $m_u$ is the atomic mass unit, as the probe object. For the speed difference between the atomic states we use $v=50$ mm $\text {s}^{-1}$ [108]. In addition, from Refs.  [108, 109] we infer that in modern atom interferometry experiments frequency shifts as small as $\Delta u_\text {min} \approx 10^{-8}$ Hz can be measured. For the dilaton’s vev we follow the ansatz made in Ref.  [50] and assume that the experiment takes place in a sufficiently small part in the center of the vacuum chamber, such that we can assume $\langle X\rangle$ to be approximately spatially constant. In addition, we only consider parts of the parameter space for which the Compton wavelength $\lambda _\textrm{C} = 1/m$ of the dilaton fulfils $\lambda _\textrm{C} \le L$, such that the field is able to take on its proper vev and not an intermediate value. In order to also consider cases for which $\lambda _\textrm{C} > L$, we would need a sufficiently good model for the evolution of the field profile from within the vacuum chamber walls to the center of the chamber, as was developed for the chameleon, for example, in Ref.  [39]. However, the sophisticated numerical studies required for this are beyond the scope of the present article.

A frequency shift is only a useful observable if we can compare it to another measurement without any frequency shift or at least with a frequency shifted by a different magnitude. Since the expression in Eq.  (36) depends on some parameters that can easily be varied, two measurements with different predicted frequency shifts can be compared. If the resulting predicted difference in frequency shifts cannot be observed, this puts constraints on the dilaton parameter space. The parameters that we choose to vary are the temperature *T* of the vacuum chamber walls, which affects the thermal part of the $\chi$-propagators, and the pressure *P*, which, if we assume that the residual gas inside the vacuum chamber has thermalized with the walls, determines the value of $\rho ^\text {ext}$ due to $\rho ^\text {ext} = P m_\text {mol}/(T R_\text {gas})$ with $m_\text {mol}$ being the molar mass of the residual gas and $R_\text {gas}$ the universal gas constant, and consequently leads to different values of $\langle X\rangle$ and *m*. For the temperature, we consider four different values: $T_1 = 0.5 \times 10^{-3}$ K, $T_2 = 100$ K, $T_3 = 300$ K, and $T_4 = 500$ K. Ref.  [44] reports a residual $\text {H}_2$ ($m_\text {mol}(\text {H}_2) = 2.016$ g/mol [110]) gas pressure of $P_2 = 9.6 \times 10^{-10}$ mbar for their experiment. However, as most extreme values, vacua with $P_1 \approx 10^{-17}$ mbar [111] can be reached, and atom interferometry experiments have been performed in warm vapors of rubidium ($m_\text {mol}(\text {Rb}) = 85.468$ g/mol [112]) atoms with pressures as high as $P_3 \approx 10^{-2}$ mbar [113].

**Fig. 3:**
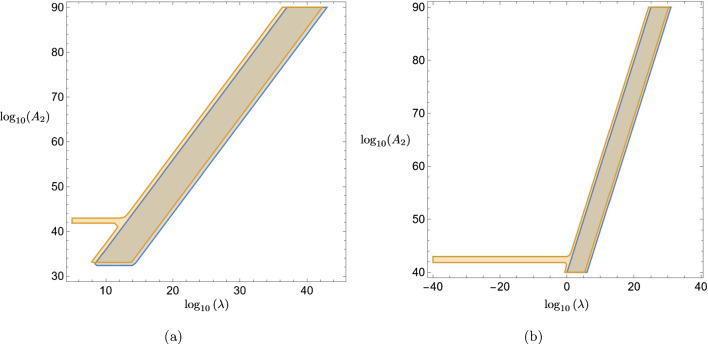
Exclusion plots for the environment dependent dilaton $(\lambda ,A_2)$-parameter space resulting from differences in frequency shifts $u(P_2,T_2;P_2,T_1)$ (blue) and $u(P_2,T_4;P_2,T_1)$ (orange); **a** $V_0 = 1\,(\text {keV})^4$, **b** $V_0 = 1\,(\text {MeV})^4$

**Fig. 4:**
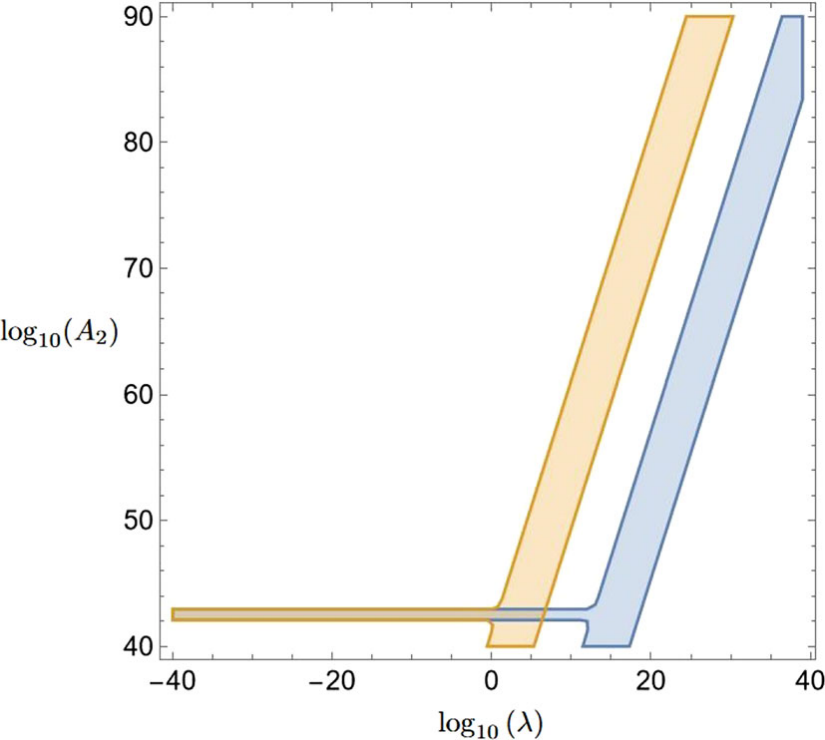
Exclusion plots for the environment dependent dilaton $(\lambda ,A_2)$-parameter space resulting from difference in frequency shifts $u(P_2,T_4;P_2,T_3)$; the blue plot represents $V_0 = 1\,\text {eV}^4$ and the orange plot $V_0 = 1\,(\text {MeV})^4$

We select two pairs of pressure and temperature, and compare the resulting difference in frequency shifts, which can only be observed if

37 $$\begin{aligned} |\Delta u(P_a,T_b) - \Delta u(P_c,T_d)|=: & {} u(P_a,T_b;P_c,T_d) \,\ge \, \Delta u_\text {min}\nonumber \\ \end{aligned}$$

is fulfilled. Furthermore, our previous assumptions require us to demand $\lambda \langle X\rangle /M_{Pl} \ll 1$ and $\sqrt{A_2} \langle X\rangle /M_{Pl} \ll 1$. Using these restrictions, and choosing values for $V_0$, we plot the parts of the $(\lambda ,A_2)$-space for which Eq.  (37) is fulfilled. The resulting plots for a few selected examples can be found in Figs.  2, and 4. In case of an experimental null result, the shaded areas in these figures exclude the respective parts of the environment dependent dilaton parameter space.

In Fig.  2 we present cases for which the lowest possible pressure $P_1$ is used, but the temperature is varied. For Fig.  2a we select $V_0 = 1\,\text {eV}^4$, and consider $u(P_1,T_2;P_1,T_1)$ (blue plot) and $u(P_1,T_4;P_1,T_1)$ (orange plot), while for Fig.  2b we use $V_0 = 1\,(\text {MeV})^4$, and keep everything else the same. Looking at these figures, we observe that in case of a null result, large parts of the dilaton parameter space could be excluded. Comparing frequency shifts with larger temperature differences, i.e.  $(T_4,T_1)$, extends the exclusion area. However, considering the lower temperature difference $(T_2,T_1)$ adds comparatively small areas to the exclusion plots, which cannot be constrained with only $(T_4,T_1)$. For the $V_0 = 1\,\text {eV}^4$ case, the horizontal exclusion area, which is more than 10 orders of magnitude in $A_2$ wide, stretches beyond $\lambda = 10^{20}$, while for $V_0 = 1\,(\text {MeV})^4$ it does not even reach $\lambda \approx 1$.

In Fig.  3 we consider $P_2$ and again vary the temperature, such that blue plots represent $u(P_2,T_2;P_2,T_1)$ and orange ones $u(P_2,T_4;P_2,T_1)$. Figure  3a depicts the case $V_0 = 1\,(\text {keV})^4$ and (b) $V_0 = 1\,(\text {MeV})^4$. Interestingly, the main difference between blue and orange shaded areas is that each orange one has a narrow (in $A_2$) horizontal section that cannot be found for the blue ones. Similarly to Fig.  2, going to higher values of $V_0$ lowers the values of $\lambda$ that can be excluded by this section. However, here, the horizontal stripe is much shorter in Fig.  3a than in b.

Finally, in Fig.  4 we again consider the pressure $P_2$, but compare frequency shifts for the highest temperatures $T_3$ and $T_4$, i.e.  we look at $u(P_2,T_4;P_2,T_3)$. The blue plot depicts the case $V_0 = 1\,\text {eV}^4$ and the orange one $V_0 = 1\,(\text {MeV})^4$. Both exclusion areas appear to be similar but shifted by more than 10 orders of magnitude on the $\lambda$-axis.

We do not find any exclusion areas for the highest pressure $P_3$ in combination with the other parameter values we chose. This can be explained by the rather large $\rho ^\text {ext}$ that results from the high pressure and temperatures in the considered warm rubidium vapors. A too dense environment suppresses the dilaton field, affecting the non-thermal part in Eq.  (36), and increases its mass, which decreases the thermal part, such that the frequency shift predicted becomes too small to be observable.

## Conclusions

Screened scalar fields are frequently used models in cosmology. Their screening mechanisms enable them to circumvent tight Solar-System constraints on their gravity-like fifth forces. However, it is possible to probe their parameter spaces in laboratory-based experiments like atom interferometry. While other screened scalar field models like the chameleon or the symmetron are already experimentally well-studied, the parameter space of the environment dependent dilaton, motivated by string theory and screened by the Polyakov–Damour mechanism, is still largely unconstrained.

Recently, Ref.  [59] presented a new path integral-based method for directly computing reduced density matrices of open quantum systems. In the present article, we applied this formalism to an open system modelled by the real, massive scalar field $\phi$ coupled to an environment comprising fluctuations $\chi$ of the dilaton field *X*. Following the study in Ref.  [50], it was our intention to use $\phi$ as proxy for an atom in matter wave interferometry. For an atom, this was a rough, but, in this particular physical situation, suitable, first approximation since the dilaton only couples to the mass density. We improved the approximation by restricting the discussion to the single-particle momentum subspace, and neglecting all diagrams containing $\phi$-loops since those would not appear at the considered perturbative order for complex composite objects like atoms. Using Ref.  [59], we computed the single-particle density matrix element in a momentum basis at final time *t* under the assumption of an initial single atom at time 0. The resulting expression was presented in Appendix A. However, for the actual discussion, we focused only on the leading effect, which turned out to be a correction to the unitary evolution of $\phi$. In an interferometry experiment, such a correction would be visible as a frequency shift if the measurement was compared to another one with a different or without any shifted frequency.

For realistic parameters of an atom interferometry experiment, we used this frequency shift in order to predict exclusion plots for the dilaton parameter space. While these predictions should be taken with care due to us approximating an atom by a scalar field, our investigation offers a glimpse at the full potential that open quantum dynamical effects have for studying and constraining physics beyond the standard models of particles and cosmology. Since the predicted exclusion plots look very promising, the present article, besides being the very first practical application of the formalism developed in Ref.  [59], serves as a strong motivation for future studies with more realistic probe systems. Those will provide us with new, powerful tools for our search after screened scalar fields and even other candidate models for dark energy or dark matter.

## Data Availability

This manuscript has no associated data or the data will not be deposited. [Authors’ comment: The only data associated with this article are the plots in Figs. 2–4, which are based on the formulas and parameter values given in Sect. 4. In case of legitimate interest, the authors will provide the Mathematica files used for producing these plots on request].

## Appendix A: Density matrix elements

Evaluating Eq.  (29) gives us

A1
